# siRNA screening identifies METTL9 as a histidine *N*π-methyltransferase that targets the proinflammatory protein S100A9

**DOI:** 10.1016/j.jbc.2021.101230

**Published:** 2021-09-23

**Authors:** Hiroaki Daitoku, Momoka Someya, Koichiro Kako, Takahiro Hayashi, Tatsuya Tajima, Hikari Haruki, Naoki Sekiguchi, Toru Uetake, Yuto Akimoto, Akiyoshi Fukamizu

**Affiliations:** 1 Life Science Center for Survival Dynamics, Tsukuba Advanced Research Alliance, University of Tsukuba, Tsukuba, Ibaraki, Japan; 2 Master's Program in Agro-Bioresources Science and Technology, Degree Programs in Life and Earth Sciences, Graduate School of Sciences and Technology, University of Tsukuba, Tsukuba, Ibaraki, Japan; 3 Faculty of Life and Environmental Sciences, University of Tsukuba, Tsukuba, Ibaraki, Japan; 4 Doctoral Program in Life and Agricultural Sciences, Degree Programs in Life and Earth Sciences, Graduate School of Sciences and Technology, University of Tsukuba, Tsukuba, Ibaraki, Japan; 5 College of Agro-Biological Resource Sciences, School of Life and Environmental Sciences, University of Tsukuba, Tsukuba, Ibaraki, Japan; 6 The World Premier International Research Center Initiative (WPI), International Institute for Integrative Sleep Medicine, University of Tsukuba, Tsukuba, Ibaraki, Japan; 7 AMED-CREST, Japan Agency for Medical Research and Development, Chiyoda-ku, Tokyo, Japan

**Keywords:** METTL9, histidine methylation, S100A9, zinc binding, cDNA, complementary DNA, ER, endoplasmic reticulum, Flag-MLCK2, Flag-tagged mouse MLCK2, GST, glutathione-*S*-transferase, HEK293T, human embryonic kidney 293T cell, METTL9-HA, hemagglutinin-tagged mouse METTL9, MLCK2, myosin light chain kinase 2, MRM, multiple reaction monitoring, MT, methyltransferase, *N*-PLA, *N*-propyl-l-arginine, PRMT, protein arginine methyltransferase, PTM, post-translational modification

## Abstract

Protein methylation is one of the most common post-translational modifications observed in basic amino acid residues, including lysine, arginine, and histidine. Histidine methylation occurs on the distal or proximal nitrogen atom of its imidazole ring, producing two isomers: *N*τ-methylhistidine or *N*π-methylhistidine. However, the biological significance of protein histidine methylation remains largely unclear owing in part to the very limited knowledge about its contributing enzymes. Here, we identified mammalian seven-β-strand methyltransferase METTL9 as a histidine *N*π-methyltransferase by siRNA screening coupled with methylhistidine analysis using LC–tandem MS. We demonstrated that METTL9 catalyzes *N*π-methylhistidine formation in the proinflammatory protein S100A9, but not that of myosin light chain kinase MYLK2, *in vivo* and *in vitro*. METTL9 does not affect the heterodimer formation of S100A9 and S100A8, although *N*π-methylation of S100A9 at His-107 overlaps with a zinc-binding site, attenuating its affinity for zinc. Given that S100A9 exerts an antimicrobial activity, probably by chelation of zinc essential for the growth of bacteria and fungi, METTL9-mediated S100A9 methylation might be involved in the innate immune response to bacterial and fungal infection. Thus, our findings suggest a functional consequence for protein histidine *N*π-methylation and may add a new layer of complexity to the regulatory mechanisms of post-translational methylation.

In eukaryotic cells, one of the most common post-translational modifications (PTMs) is protein methylation, that is, the transfer of a methyl group from SAM to lysine and arginine residues in the target protein (1, 2, 3, 4). Lysine and arginine methylation consist of three distinct forms: monomethylation, dimethylation, and trimethylation of lysine and monomethylation, disymmetric methylation, and diasymmetric methylation of arginine. The functional significance of protein methylation has been extensively studied in histones, whose methylation plays a fundamental role in regulating gene expression and chromatin state (5, 6). Recently, however, a growing body of evidence has established that methylation also occurs on nonhistone proteins and is involved in a widespread phenomenon that regulates diverse biological processes, including protein synthesis, mRNA splicing, and signal transduction (7, 8). In addition to lysine and arginine, protein methylation of atypical residues, such as glutamine and histidine, is considered to have profound biochemical and physiological significance in eukaryotes (9, 10).

Histidine can potentially be methylated on the nitrogen in either position 1 (*N*π) or 3 (*N*τ) of the imidazole ring, producing the isomers *N*π-methylhistidine (also referred to as 1-methylhistidine) or *N*τ-methylhistidine (3-methylhistidine), respectively (9). Protein histidine methylation was first discovered as *N*τ-methylhistidine in the constituents of actin and myosin from muscle proteins half a century ago (11, 12). After that, histidine methylation has been known for many years, but so far, there are only a few reports regarding histidine-methylated proteins: yeast ribosomal protein Rpl3 that carries an *N*τ-methylhistidine (13), mammalian myosin light chain kinase 2 (MLCK2) (14), and proinflammatory protein S100A9 (15), both of which carry an *N*π-methylhistidine. However, a recent high-throughput proteomics analysis has successfully captured hundreds of proteins methylated at histidine residue in human cells, indicating that protein histidine methylation may be more prevalent type of PTM than predicted (16).

The biggest obstacle to understanding the biological significance of histidine methylation is the lack of information about enzymes that catalyze methylhistidine formation. At present, however, the two distinct histidine-specific protein methyltransferases (MTases) have been identified in yeast and mice. The first one is yeast histidine protein MTase 1 that belongs to the seven-β-strand MTases responsible for the *N*τ-methylation of His-243 in Rpl3 (13), thereby contributing to the assembly of the large ribosomal subunit and translational elongation fidelity (17, 18). The second one is mouse SETD3 that belongs to the SET domain MTases responsible for the *N*τ-methylation of His-73 in the actin and regulates contraction in uterine smooth muscle (19, 20). It should be noted that both enzymes are histidine *N*τ-MTases, and there are no reports on enzymes that catalyze histidine *N*π-methylation so far.

In this study, we established a new method that allows discrimination of the methylhistidine isomers of cellular proteins by using LC–MS/MS and combined it with an unbiased siRNA screen to identify molecules specifically required for histidine *N*π-methylation of S100A9. We found that mammalian seven-β-strand MTase METTL9 introduces *N*π-methylhistidine in S100A9, but not MLCK2, *in vivo* and *in vitro*. We also observed that METTL9 is localized predominantly to the endoplasmic reticulum (ER), binding to and methylating S100A9. Furthermore, we demonstrated that METTL9-mediated methylation of S100A9 at His-107 did not affect its heterodimer formation with S100A8, whereas histidine methylation of recombinant S100A9 by METTL9 substantially decreases its binding affinity to zinc. Thus, our present findings show that METTL9 is a *bona fide* histidine *N*π-MTase in the ER and contributes to histidine *N*π-methylation of S100A9, reducing its zinc-binding ability. We believe that these findings have broad implications for not only the molecular basis of protein histidine methylation but also the biological function of METTL9.

## Results

### Identification of METTL9 as a protein histidine MTase targeting S100A9 *in vivo*

**Figure 1:**
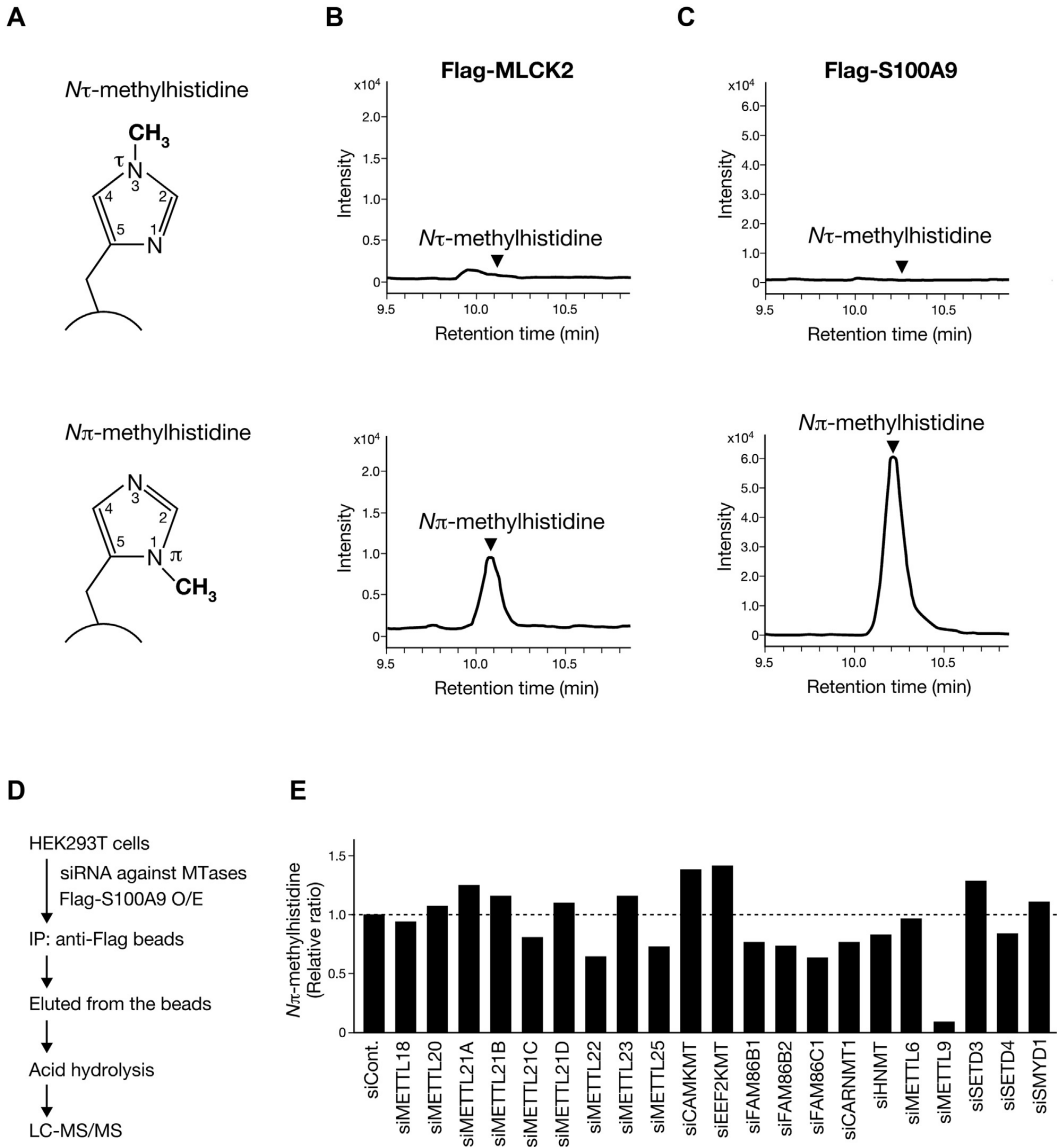
**METTL9 is a protein histidine *N*π-methyltransferase that methylates S100A9.***A*, histidine methylation: *top*, *N*τ-(3)-methylhistidine, *bottom*, *N*π-(1)-methylhistidine. A *circular arc* indicates the polypeptide backbone. *B* and *C*, both cellular MLCK2 and S100A9 contain *N*π-methylhistidine but not *N*τ-methylhistidine. Flag-MLCK2 (*B*) and Flag-S100A9 (*C*) transiently expressed in HEK293T cells were immunopurified and then subjected to acid hydrolysis. Their methylhistidine contents were determined by LC–MS/MS and shown as chromatograms. *Arrowheads* indicate the retention time of the *N*τ-methylhistidine and *N*π-methylhistidine defined by their standards. *Top*, *N*τ-methylhistidine and *bottom*, *N*π-methylhistidine. *D*, schematic of biochemical strategy to identify an enzyme that methylates Flag-S100A9 in HEK293T cells. *E*, siRNA screening for an enzyme that catalyzes histidine *N*π-methylation of Flag-S100A9. HEK293T cells transfected with siRNAs as indicated were further transfected with Flag-S100A9 expression plasmid, followed by immunopurification and acid hydrolysis. Their methylhistidine contents were determined by LC–MS/MS and shown as a relative ratio compared with control siRNA knockdown. HEK293T, human embryonic kidney 293T cell; IP, immunoprecipitation; MLCK2, myosin light chain kinase 2; O/E, overexpression.

Histidine methylation can occur on the distal or proximal nitrogen atom of its imidazole ring, producing *N*τ-methylhistidine or *N*π-methylhistidine, respectively (Fig. 1*A*). To identify the protein histidine MTase that catalyzes *N*π-methylhistidine *in vivo*, we developed a novel method to distinguish either the *N*τ or *N*π histidine methylation of cellular proteins. First, a protein of interest is ectopically expressed as Flag-tagged forms in human embryonic kidney 293T (HEK293T) cells and then immunopurified with Flag affinity resin, followed by acid hydrolysis and subsequent analysis of the resulting amino acids by LC coupled to tandem MS (LC–MS/MS). To verify the validity of this method, we selected two mammalian proteins, MLCK2 and S100A9, both of which are known to contain *N*π-methylhistidine residues (14, 15), and performed the aforementioned experiment. Total protein hydrolysates of Flag-tagged mouse MLCK2 (Flag-MLCK2) showed a strong peak at the retention time of *N*π-methylhistidine, whereas no peak was observed for *N*τ-methylhistidine (Fig. 1*B*). Similar results were observed in Flag-tagged mouse S100A9 (Flag-S100A9) protein, but it should be noted that the signal intensity of *N*π-methylhistidine was increased 6-fold when compared with that of Flag-MLCK2 protein (Fig. 1*C*). These data suggest the presence of a protein histidine *N*π-MTase(s) that targets MLCK2 and/or S100A9 in HEK293T cells and led us to identify the endogenous enzyme(s) using an siRNA screening approach.

To this end, we adopted a Flag-S100A9 protein as a target substrate for enzyme screening because of its higher methylation efficiency than MLCK2. We prepared siRNA duplexes against the previously uncharacterized MTase genes, focusing on the sequence homology to yeast Hpm1 and mouse SETD3. As depicted in Figure 1*D*, HEK293T cells were transfected with siRNA pools targeting individual genes together with Flag-S100A9 expression plasmid, and then immunopurified Flag-S100A9 proteins were analyzed by LC–MS/MS. Of the 21 candidate genes, only METTL9 knockdown resulted in a marked decrease in the amount of *N*π-methylhistidine of S100A9, following the inhibition of *METTL9* gene expression (Figs. 1*E* and S1). In contrast, knockdown of either SETD3 or METTL18, a putative human ortholog of yeast Hpm1, showed no noticeable change in the *N*π-methylhistidine levels of S100A9 (Fig. 1*E*). In this way, our screening approach identified METTL9 as a novel protein histidine MTase that specifically catalyzes the *N*π-methylhistidine formation of S100A9 *in vivo*.

### METTL9 directly methylates S100A9 at His-107 *in vitro*

**Figure 2:**
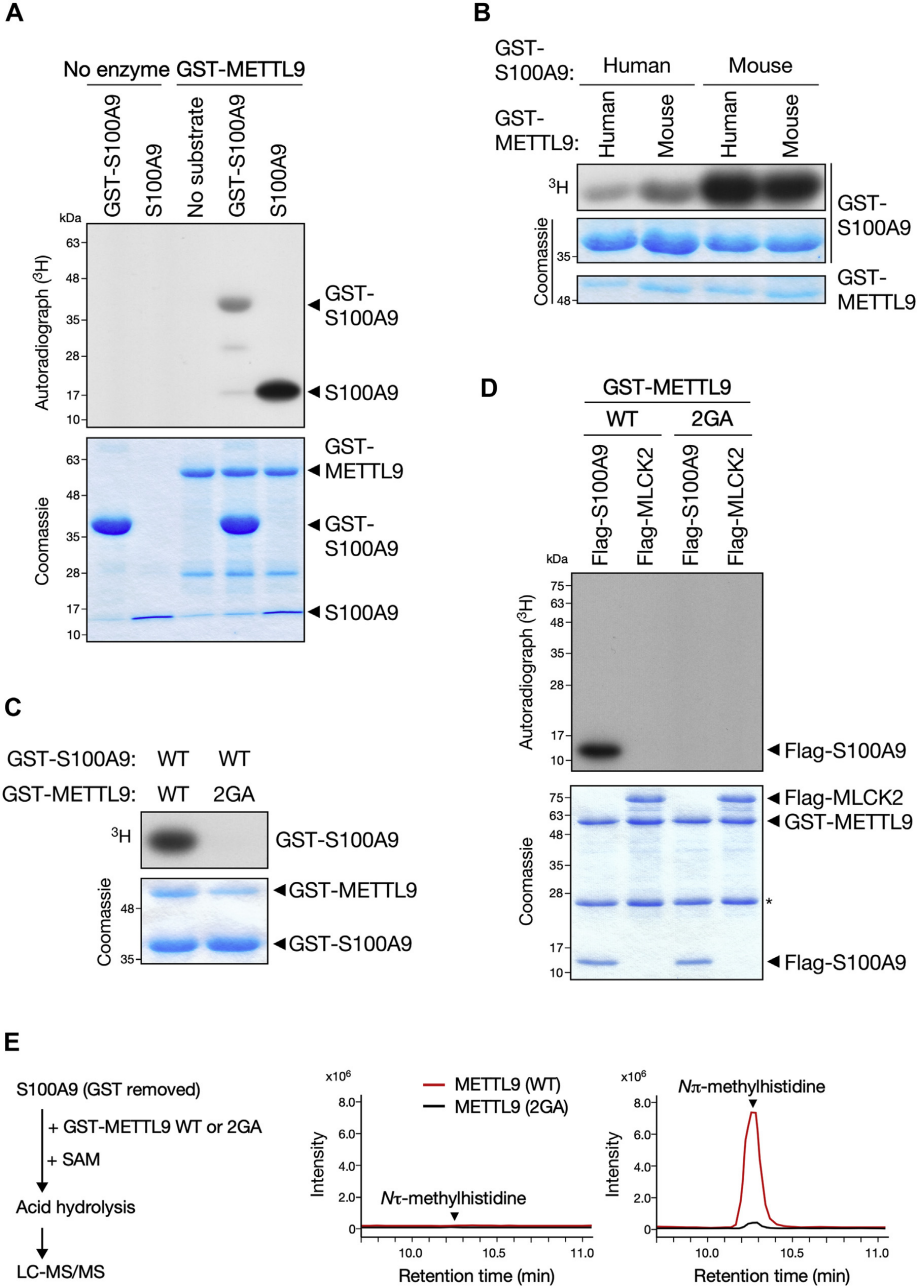
**METTL9 directly methylates S100A9 at His-107.***A*, recombinant METTL9 methylates S100A9. Glutathione-*S*-transferase (GST)-fused or GST-cleaved S100A9 (mouse) were incubated with ^3^H-SAM in the presence or the absence of GST-METTL9 (human). The incorporation of [^3^H]-methyl into proteins was visualized by autoradiography (*top*). Coomassie blue stain of proteins used in the reaction were shown as a loading control (*bottom*). *B*, METTL9 preferentially methylates mouse S100A9. *In vitro* methylation reactions as in (*A*) with GST-fused human or mouse METTL9 together with GST-fused human or mouse S100A9 as the substrate. *C*, METTL9 2GA mutant fails to methylate S100A9 *in vitro*. *In vitro* methylation reactions with GST-S100A9 together with GST-METTL9 WT or 2GA mutant. *D*, METTL9 does not methylate MLCK2. *In vitro* methylation reactions with GST-METTL9 WT or 2GA mutant together with immunopurified Flag-S100A9 or Flag-MLCK2 as the substrates. The *asterisk* indicates IgG light chain. *E*, METTL9 catalyzes *N*π-methylhistidine formation on S100A9 *in vitro*. GST-cleaved S100A9 was incubated with GST-METTL9 WT or 2GA mutant in the presence of SAM. After acid hydrolysis, methylhistidine contents were determined by LC–MS/MS and shown as chromatograms. *Arrowheads* indicate the retention time of the *N*τ-methylhistidine and *N*π-methylhistidine defined by their standards.

To test whether METTL9 directly methylates S100A9, we performed an *in vitro* methylation assay by measuring the incorporation of the [^3^H]-methyl group of [^3^H]-SAM into recombinant glutathione-*S*-transferase (GST)-fused S100A9. We detected the methylation signal of GST-S100A9 by fluorography only when incubated with GST-METTL9, and the signal was greatly enhanced against GST-cleaved S100A9 (Fig. 2*A*). Since we identified the “human” METTL9 gene as the responsible enzyme for histidine *N*π-methylation of “mouse” S100A9, we evaluated the species specificity of S100A9 methylation between humans and mice. We found that mouse S100A9, originally reported to contain *N*π-methylhistidine residue (15), is a more favorable substrate than human ortholog, whereas the enzymatic activities of METTL9 are similar (Fig. 2*B*). Thus, we investigated the molecular function of mouse METTL9 in the methylation of mouse S100A9. First, we generated an MTase-inactive mutant of METTL9 by alanine substitution of Gly-153 and Gly-155 in the glycine-rich SAM-binding motif (referred to as 2GA) and showed that the 2GA mutant failed to methylate S100A9 (Fig. 2*C*). Since mouse S100A9 has been reported to be methylated at His-107 (15), we replaced His-107 of S100A9 with phenylalanine and confirmed that this H107F mutation abolished METTL9-mediated methylation (Fig. S2). Next, to examine the substrate specificity of METTL9, we immunopurified Flag-tagged MLCK2 proteins from HEK293T cells and used them in an *in vitro* methylation assay. In contrast to S100A9, MLCK2 was not *de novo* methylated by METTL9, suggesting that S100A9 is a specific target of METTL9 (Fig. 2*D*). To further obtain evidence for histidine *N*π-MT activity of METTL9 *in vitro*, we employed LC–MS/MS to analyze recombinant S100A9 proteins, which were incubated with GST-METTL9 WT or 2GA mutant in the presence of nonradioisotope-labeled SAM. Consistent with the results of the siRNA screening (Fig. 1*E*), METTL9 selectively introduced *N*π-methylhistidine in S100A9 *in vitro* (Fig. 2*E*). Taken together, our data established METTL9 as a *bona fide* protein histidine *N*π-MT that directly methylates S100A9 at His-107.

### The N-terminal sequence of METTL9 is not required for its subcellular localization and S100A9 methylation

**Figure 3:**
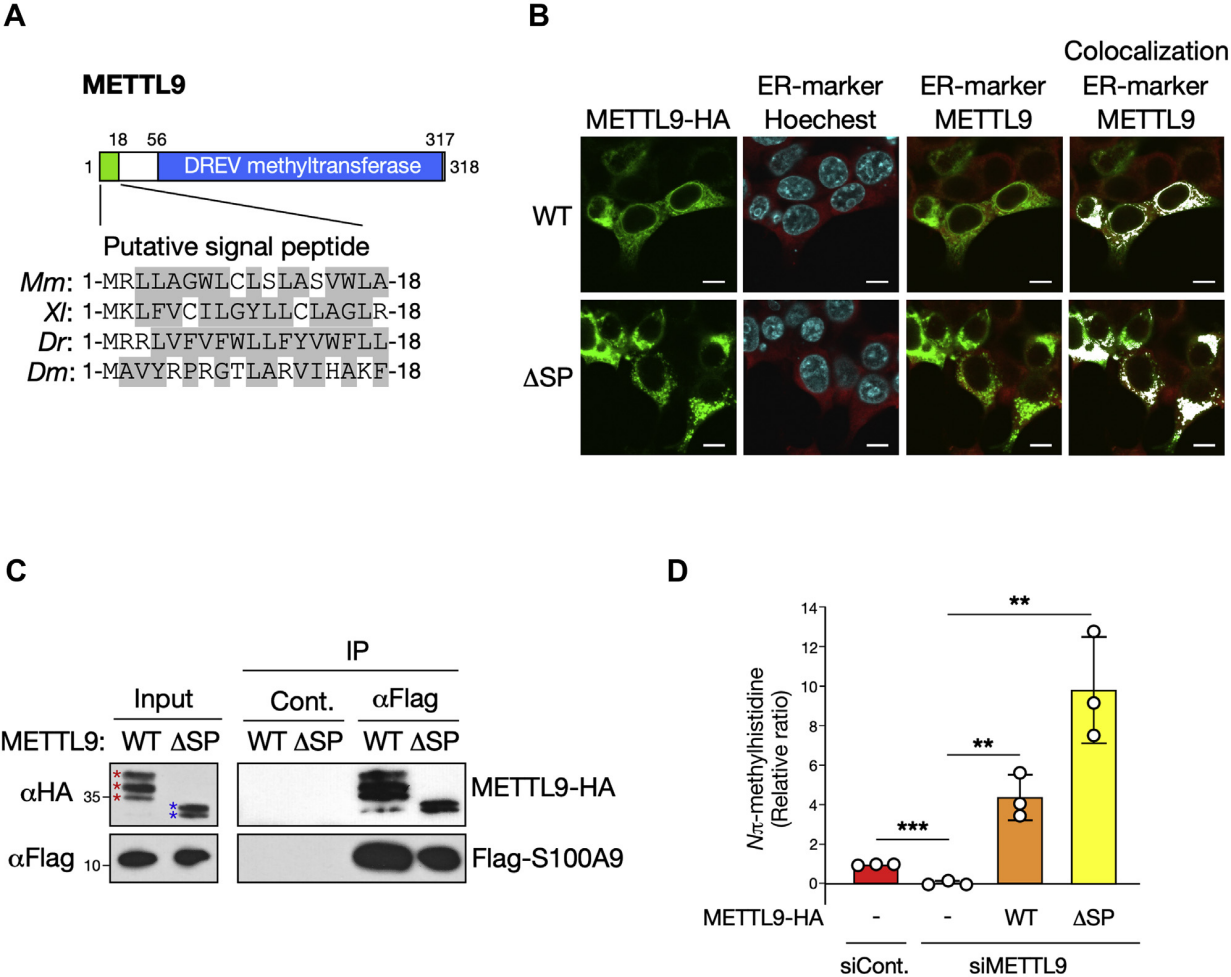
**The N-terminal sequence of METTL9 is not involved in its localization and S100A9 methylation.***A*, *top*, domain structure of METTL9 containing a putative signal peptide at N terminus (*green*) and a C terminus homologous to DREV methyltransferase (*blue*). *Bottom*, sequence alignment of the N-terminal end of METTL9 orthologs across species. Hydrophobic amino acids are denoted by *shaded gray*. *B*, METTL9 localizes to the endoplasmic reticulum (ER). Representative immunofluorescence images of METTL9 WT (*top*) or ΔSP mutant (*bottom*) localization in HEK293T cells. *Green*, METTL9-HA; *red*, ER marker (Alexa594-conjugated concanavalin A); *blue*, nucleus (Hoechst 33258). The scale bar represents 10 μm. Images of ER marker are merged with Hoechst and METTL9-HA staining, respectively. Colocalization indicates overlay of ER marker and METTL9-HA, shown as *white pixels*. *C*, METTL9 ΔSP mutant can bind to S100A9 in cells. Coimmunoprecipitation of Flag-S100A9 with METTL9-HA WT and ΔSP mutant is shown by Western blot. Input indicates 5% of whole-cell lysates used for immunoprecipitation. *Colored asterisks* indicate the bands corresponding to METTL9-HA WT (*red*) and ΔSP mutant (*blue*). Normal mouse IgG was used as a negative control of immunoprecipitation. *D*, complementation of METTL9 knockdown cells with METTL18 ΔSP mutant as well as WT restores *N*π-methylhistidine levels of S100A9. HEK293T cells transfected with control or METTL9 siRNAs were further transfected with Flag-S100A9 together with or without METTL9-HA expression plasmids as indicated. *N*π-methylhistidine levels of Flag-S100A9 are shown as a relative ratio to control knockdown. Mean ± SD (*n* = 3 independent experiments). ∗∗∗*p* < 0.001; ∗∗*p* < 0.005; two-tailed Student's *t* test. *Dr*, *Danio rerio*; *Dm*, *Drosophila melanogaster*; *Mm*, *Mus musculus*; HEK293T, human embryonic kidney 293T cell; *Xi*, *Xenopus laevis.*

In addition to the classical 7BS MTase feature motif designated as DREV, METTL9 possesses a putative N-terminal signal peptide (residues 1–18) that often targets the nascent protein to the lumen of the ER (Fig. 3*A*) (21). We therefore observed the subcellular localization of METTL9 by immunofluorescence staining for a C-terminal hemagglutinin-tagged mouse METTL9 (METTL9-HA) with a fluorescent marker for the ER (concanavalin A-conjugated Alexa Fluor 594). As expected, we found that METTL9-HA was localized mainly in the ER in both HEK293T and HeLa cells (Figs. 3*B* and S3, *top*). Unexpectedly, however, a deletion mutant lacking the signal peptide (hereafter, called METTL9ΔSP-HA) exhibited a partially aggregated but nearly identical distribution to that of the WT (Figs. 3*B* and S3, *bottom*). This observation implies that the ER localization of METTL9 occurs independently of its N-terminal sequence and led us to examine the possible functions of the N-terminal sequence. Thus, we first tested the interaction between METTL9ΔSP-HA and Flag-S100A9 in HEK293T cells. The coimmunoprecipitation assay showed that deletion of the N-terminal sequence of METTL9 appears not to influence its binding to S100A9 (Fig. 3*C*). To further reinforce the notion that the N-terminal sequence of METTL9 is not involved in the regulation of S100A9, we determined whether the METTL9ΔSP mutation affects the S100A9 methylation *in vivo*. We knocked down endogenous METTL9 in HEK293T cells and subsequently cotransfected with either WT or the ΔSP mutant of mouse METTL9-HA together with Flag-S100A9 expression plasmids. LC–MS/MS analysis demonstrated that, the *N*π-methylhistidine level of S100A9 was virtually abolished by METTL9 knockdown, whereas complementation of the knockdown cells with the METTL9ΔSP mutant and WT actually restored *N*π-methylhistidine to levels higher than those observed in S100A9 from control knockdown cells (Fig. 3*D*). In conclusion, these results indicate that the N-terminal sequence of METTL9 does not significantly contribute to its ER localization and methylation of S100A9 in cells.

### METTL9-mediated methylation of S100A9 affects no heterodimer formation but zinc binding

**Figure 4:**
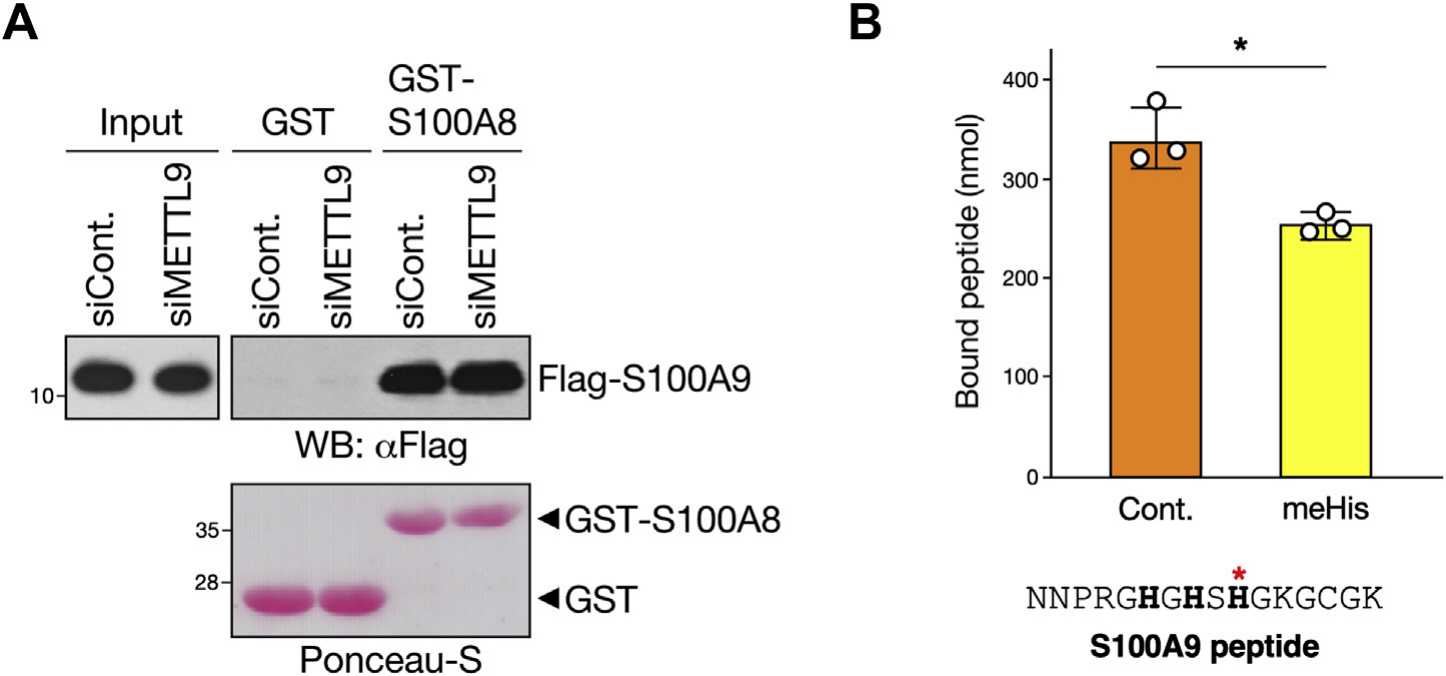
**METTL9-mediated methylation of S100A9 decreases the binding affinity to zinc.***A*, METTL9-mediated methylation of S100A9 does not affect the dimerization between S100A9 and S100A8. HEK293T cells transfected with control or METTL9 siRNAs were further transfected with Flag-S100A9, the whole-cell lysates were subjected to GST pull-down assay with GST alone or GST-fused S100A8, followed by Western blot using anti-Flag antibody (*top*). Input indicates 5% of whole-cell lysates used for the GST pull-down assay. Ponceau-S staining of GST proteins used in the reaction was shown as a loading control (*bottom*). *B*, histidine *N*π-methylation of S100A9 peptide attenuates its binding to zinc. A synthetic S100A9 peptide containing either none (control) or 1 *N*π-methylhistidine (meHis) residue was passed through a zinc-chelating column, and then, the bound peptide was measured by LC–MS/MS. The peptide sequence used in this assay is shown, and the methylated histidine corresponding to His-107 of S100A9 is indicated with a *red star*. Mean ± SD (*n* = 3 independent experiments). ∗*p* < 0.05 (*p* = 0.0124); two-tailed Student's *t* test. GST, glutathione-*S*-transferase; HEK293T, human embryonic kidney 293T cell.

Finally, we attempted to uncover the functional significance of METTL9-mediated S100A9 methylation. Since S100A9 is known to form a heterodimeric complex with S100A8 through the EF-hand motifs, a central calcium-binding loop flanked by two a-helices (Fig. S4) (22), we first examined the possibility that METTL9-mediated methylation may affect the formation of S100A8/S100A9 heterodimer *in vivo*. To this end, HEK293T cells expressing Flag-S100A9 were transfected with or without siRNA against METTL9, and their whole-cell lysates were used for pull-down assays using GST-fused mouse S100A8 (GST-S100A8). We showed that siRNA ablation of METTL9 did not affect the interaction between S100A9 and S100A8, despite a substantial decrease in the methylation levels of S100A9 (Figs. 4*B* and S5). To further confirm this result, we prepared recombinant S100A9 by cleavage of GST-S100A9, followed by an *in vitro* methylation reaction with GST-METTL9 in the presence or the absence of SAM, and then evaluated the binding affinity to GST-S100A8. In agreement with the results using cell lysates, we found that the S100A9–S100A8 interaction was not altered by *de novo* methylation (Fig. S6). Collectively, we concluded that *N*π-methylation of S100A9 at His-107 did not influence the heterodimerization of S100A8–S100A9.

In addition to the Ca^2+^-binding EF-hand motifs involved in heterodimer formation, S100A8 and S100A9 are structurally characterized by a Zn^2+^-binding site that consists of the HxxxH motif (Fig. S4) (22, 23). Mouse S100A9 contains two HxxxH motifs close to the C terminus. Interestingly, the second overlaps with the histidine residue that undergoes *N*π-methylation (Fig. S4): this prompted us to investigate whether METTL9-mediated methylation of S100A9 influences its zinc-binding ability. To this end, we synthesized an S100A9-derived peptide containing the second HxxxH motif (residues 98–113), as well as a methylated version of this peptide at His-107, and then compared their zinc-binding activity using metal-chelate chromatography on iminodiacetic acid equilibrated with zinc. Interestingly, only one histidine *N*π-methylation of the S100A9 peptide resulted in a significant reduction in zinc binding, suggesting that METTL9-mediated methylation modulates the zinc-binding ability of S100A9 (Fig. 4*B*).

## Discussion

In this study, we discovered a new histidine *N*π-MT, METTL9, using siRNA screening followed by quantitative analysis of methylhistidine. This method is based on the chemical properties of protein methylation; unlike phosphorylation and acetylation, methyl amino acids, including methyl-histidine, methyl-lysine, and methyl-arginine, are stable even under acid hydrolysis conditions (6 N HCl at 110 °C for 24 h). LC–MS/MS analysis of the methylhistidine isomers enabled us to distinguish between *N*τ histidine methylation and *N*π histidine methylation of cellular proteins. Because there are no available antibodies that specifically recognize protein histidine methylation, our approach could be a gold standard for assessing the methylation status of METTL9 substrates as well as screening novel protein histidine MTase(s).

In preparation of this article, Davydova *et al*. (24) have first reported that METTL9 is a broad-specificity MT that introduces *N*p-methylhistidine at His-x-His (where x is optimally a small residue) motifs in several different proteins. Thereafter, Lv *et al*. (25) also provided evidence for METTL9-mediated *N*p-methylhistidine formation and confirmed that METTL9 recognizes an x-His-x-His motif in the substrate protein. However, while their focuses were on METTL9 as one of the uncharacterized mammalian 7BS MTases, our present work set out to identify the enzymes responsible for histidine *N*p-methylation of S100A9 by using an unbiased siRNA screen (Fig. 1*D*). Notably, this approach allows exploring a novel MTase that catalyzes histidine methylation of previously reported protein, such as MLCK2 and myosin heavy chain (14). Because we have successfully detected *N*p-methylhistidine in immunopurified Flag-MLCK2 in HEK293T cells (Fig. 1*B*), it would be possible to obtain an MLCK2-histidine Nπ-methyltransferase from a genome-wide MTase siRNA library.

We observed that METTL9-HA was localized exclusively in the ER in both HeLa and HEK293T cells (Figs. 3*B* and S2, *top*). Although it seems likely that subcellular localization of METTL9 was determined by a putative N-terminal signal peptide conserved across species, ablation of the sequence did not alter its ER localization, albeit more dot-like distribution than that of the WT (Figs. 3*B* and S2, *bottom*). Moreover, since the METTL9ΔSP mutant could bind and methylate S100A9 in cells, it remains unclear whether the N-terminal sequence plays a functional role in METTL9. Instead, we noted the potential PTMs of METTL9. As shown in Figure 3*C*, the immunoblot patterns of METTL9-HA showed an upper band shift above its estimated molecular size of 35 kDa, whereas no band shift was observed when the ΔSP mutant was expressed. Considering that the ER is an organelle in which N-linked glycosylation occurs (21), the upper band shift may indicate N-linked glycosylation of METTL9. The N-terminal sequence appears to be required for this modification. Supporting this idea, METTL9 includes the putative N-linked glycosylation consensus motif Asn–x–Ser/Thr (where x can be any amino acid but proline) (21). Further investigation is needed to determine the significance of the N-terminal sequence in the METTL9 function.

Our *in vitro* experiments demonstrated that *N*π-methylation at His-107 of S100A9 peptide resulted in the attenuation of zinc binding (Fig. 4*C*). This observation indicates that METTL9-mediated methylation modulates the zinc-binding activity of S100A9. The S100A8–S100A9 heterodimer, also called as calprotectin, is known to be abundant in the cytosol of circulating neutrophils and plays a prominent role in the bactericidal action through two regulatory mechanisms *via* zinc binding (26). The first is an extracellular function of S100A8–S100A9, which exerts an antimicrobial activity, presumably by chelation of zinc, essential for the growth of bacteria and fungi (27, 28, 29). The second is an intracellular function of zinc-induced (S100A8–S100A9) 2-tetramers, which have been shown to modulate tubulin polymerization and stabilization during migration of phagocytes (30). Thus, METTL9-mediated S100A9 methylation appears to be involved in the innate immune response to bacterial infection. In support of this hypothesis, the RNA-Seq datasets from the ImmGen project show that the expression of *METTL9* as well as S100A9 is extremely high in neutrophils (31). Although the *Mettl9* KO mice were reported to exhibit no obvious phenotype (24), more detailed studies on the innate immune response could shed light on the biological function of METTL9.

We found that METTL9 catalyzes *N*π-methylhistidine formation in S100A9, whereas no *N*τ-methylhistidine was generated even when reacted with excess amounts of METTL9, S100A9, and SAM *in vitro* (Fig. 2*E*). In contrast, previously known histidine MTases, yeast Hpm1, and mouse SETD3 have been shown to catalyze only *N*τ-methylhistidine formation in Rpl3 and actin, respectively (13, 19, 20). These strict specificities appear to be analogous to arginine methylation by the protein arginine methyltransferase (PRMT) family members; namely, type I enzymes (*e.g.*, PRMT1) catalyze the production of asymmetrically dimethylated arginine, whereas type II enzymes (*e.g.*, PRMT5) catalyze the production of symmetrically dimethylated arginine (32). Given the aforementioned facts, it is plausible that the seemingly slight difference in which the position of the nitrogen is methylated in His/Arg residues has important implications for the modulation of modified protein function. Structural analyses of the catalytic mechanisms underlying the recognition and methylation of S100A9 by METTL9 are required to verify this assumption. Comparison of the METTL9 structure with that of SETD3 will provide insights into the protein histidine methylation phenomenon (33). Immune response could shed light on the biological function of METTL9.

The biological function of METTL9 has yet to be revealed; however, our data point out a possible link between METTL9 activity and innate immune response to bacterial infection through histidine methylation of S100A9. Notably, an integrated database of human maladies and their annotations (MelaCards) associates *METTL9* with inflammatory bowel disease one that is characterized by a chronic relapsing intestinal inflammation. Thus, understanding the contribution of METTL9-mediated histidine methylation of S100A9 in particular to immune response holds the potential of identifying new therapeutic targets and developing alternative treatment strategies for bacterial infections.

## Experimental procedures

### Gene cloning and mutagenesis

Coding sequences were amplified with PrimeSTAR MAX DNA polymerase (Takara Bio) from mouse spleen, heart, or HEK293 complementary DNAs (cDNAs) and were inserted into pcDNA3 vector with N-terminal Flag tag (mouse MLCK2 and mouse S100A9), pcDNA3 vector with C-terminal HA tag (mouse METTL9), or pGEX-6P-1 vector (mouse/human S100A9, mouse/human METTL9, and mouse S100A8). Deletion and point mutations were generated by PCR-based mutagenesis using specific primers. All constructs were verified by sequencing.

### Reagents

*N*π-methylhistidine, *N*τ-methylhistidine, and *N*-propyl-l-arginine (*N*-PLA) were obtained from Sigma–Aldrich. Peptides derived from S100A9 (amino acids 98–113) were synthesized containing either nonmethylhistidine or *Nπ*-methylhistidine at His-107 (Sigma–Aldrich). The following antibodies were used in this study: Flag (MBL), HA (Sigma–Aldrich), antimouse peroxidase-conjugated antibody (Cytiva), Alexa488 antimouse antibody (Life Technologies), and anti-FLAG M2 affinity gel (Sigma–Aldrich). Western blots were visualized by chemiluminescence using Clarity Western ECL substrate (Bio-Rad).

### Cell culture, transfection, and siRNA

HEK293T and HeLa cells were cultured in Dulbecco's modified Eagle's medium supplemented with 10% fetal bovine serum (Gibco) and 1% penicillin–streptomycin solution Hybri-Max (Sigma–Aldrich). Transfection of plasmid DNA and siRNA duplexes was performed using GeneJuice Transfection Reagent (Novagen) and Lipofectamine RNAiMAX (Invitrogen), respectively, according to the manufacturer's manuals. The ON-TARGET*plus* SMART pool siRNA duplexes used in the siRNA screening were purchased from Horizon and listed in Table S1.

### Expression and purification of recombinant proteins

GST-tagged hMETTL9, mMETTL9, mMETTL9 G153A/G155A (2GA), hS100A9, mS100A9, mS100A9 H107F, and mS100A8 were expressed in *Escherichia coli* and batch purified by an affinity chromatography resin. In brief, the appropriate pGEX-6P-1 constructs were transformed into the chemically competent BL21-CodonPlus (DE3)-RIL (Agilent Technologies) *E. coli* strain. The bacteria were cultured in LB medium with ampicillin until the absorbance reached 0.8 at 600 nm, and subsequently, protein expression was induced with 0.5 mM IPTG at 22 °C for 3 h. After incubation in 1× PBS supplemented with 1 mg/ml lysozyme on ice for 10 min, the bacteria were lysed by sonication in 0.5% Nonidet P-40, 1 mM DTT, and 1× Protease Inhibitor Cocktail (Nacalai Tesque, Inc), and then GST-tagged proteins were purified using Glutathione Sepharose 4B (GE Healthcare). For GST-tag removal, PreScission Protease was added to GST-tagged protein–bound glutathione beads in cleavage buffer (50 mM Tris–HCl, pH 8.0, 150 mM NaCl, 1 mM EDTA, and 1 mM DTT), and the reactions were incubated with vortex mixing at 4 °C for 4 h.

### Methylhistidine content analysis of immunopurified proteins

HEK293T cells expressing Flag-S100A9 or Flag-MLCK2 were lysed in cell-lysis buffer (20 mM Hepes–KOH, pH 7.9, 150 mM NaCl, 0.1% Triton X-100, and 0.1 mM EDTA) supplemented with 1× Protease Inhibitor Cocktail (Nacalai Tesque, Inc), followed by immunoprecipitation with ANTI-FLAG M2 Affinity Gel for 3 h at 4 °C. After washing with the same buffer for three times, Flag-tagged proteins were eluted with gentle shaking in 0.1 M glycine HCl (pH 2.5) buffer for 5 min at 25 °C, and the beads were centrifuged at 5000*g* for 1 min. The supernatants were transferred to dialysis cups (BMBio), which had a molecular weight cutoff of 3500 Da, and were dialyzed against PBS buffer for 6 h and further dialyzed against pure water for 18 h at 4 °C. The dialysates were analyzed by LC–MS/MS as described later.

### Estimation of histidine methylation on proteins by LC–MS/MS

Samples were separated using a Nexera Ultra High-Pressure LC System (Shimadzu). For the separations of histidine derivatives and *N*-PLA, as an internal standard, the protein samples (obtained by immunopurified or recombinant) spiked with 25 ng of *N*-PLA were hydrolyzed with 6 N HCl at 110 °C for 24 h, and the hydrolysate was dissolved in HPLC-grade purified water. About 10 μl was injected onto a SeQuant ZIC-HILIC column (2.1 × 150 mm; Merck KGaA) with a SeQuant TM ZIC-HILIC Guard Fitting (1.0 × 14 mm; Merck KGaA) kept at 40 °C. Mobile phases A and B were prepared as the ratios of water:acetonitrile:formic acid were 98:1:1 and 1:98:1, respectively. Gradient elution was performed at 0.2 ml/min using mobile phase A and B as follows: 0 to 1.0 min isocratic 95% B; 1.0 to 10.0 min linear gradient from 95 to 5% B; 10.0 to 14.0 min isocratic 5% B; 14.0 to 14.1 min linear gradient from 5 to 95% B; and column re-equilibration from 14.1 to 21.0 min at 95% B.

Detection of analyte was performed by an LCMS-8050 triple quadrupole mass spectrometer (Shimadzu) in multiple reaction monitoring (MRM) mode, using positive electrospray ionization. After *N*π-methylhistidine and *N*τ-methylhistidine ions with identical masses (*m/z* 170.1) pass through the first mass filter (Q1) in the MS apparatus, fragment ions with different molecular weights (*m/z* 96.1 and *m/z* 124.1, respectively) are generated during Ar gas collision-induced dissociation because of the different bonding positions of the methyl groups. To quantify *N*π-methylhistidine and *N*τ-methylhistidine separately, these fragment ions were detected with the following mass filter (Q3). The transitions based on the MRM of histidine, *N*π-methylhistidine, *N*τ-methylhistidine, and *N*-PLA were determined as *m/z* 156.1 > 110.1, 170.1 > 96.1, 170.1 > 124.1, and 216.60 > 70.15, respectively.

Besides ion spray source at 300 °C in the positive mode with unit resolution for Q1 and Q3, other optimized parameters are as follows; interface voltage of 4.0 kV, interface current of 0.1 μA, conversion dynode potential of 10 kV, detector potential of 2.44 kV, nebulizer gas flow rate of 3 l/min, heating gas flow rate of 10 l/min, drying gas flow rate of 10 l/min, collision gas (Ar) of 270 KPa, desolation line temperature of 250 °C, and heat block temperature of 400 °C. System operation and data acquisition and processing were completed with the LabSolutions for LC–MS, version 5.60 software (Shimadzu). A series of standard samples were prepared and run simultaneously with each experimental sample. Calibration curves were constructed based on the data of concentration *versus* peak area using the dilution series for histidine derivatives (histidine, *N*π-methylhistidene, and *N*τ-methylhistidene) and *N*-PLA in the ranges of 0.1 to 10 pmol. The observed concentrations and percentages of the analytes in each experiment were calculated from the calibration curves.

### Quantitative analyses of peptides using LC–MS/MS

Analyzing unmodified and *N*π-methylated peptide at His-107 of mS100A9 (98–112) was carried out using the Shimadzu LC–MS/MS system. The eluents from HiTrap chelating column were 1.0 × 10^4^ times diluted with HPLC-grade purified water, and 10 μl was injected onto an Inertsil ODS-HL column (3 mm, 2.1 × 150 mm; GL Science) with an Inertsil ODS-HL Cartridge Guard Column (3 mm, 3.0 × 10 mm; GL Science) kept at 30 °C. Mobile phases A and B were 0.1% formic acid aqueous solution and 0.1% formic acid in acetonitrile, respectively. Gradient elution was performed at 0.2 ml/min using mobile phase A and B as follows: 0 to 5.0 min isocratic 0% B; 5.0 to 15.0 min linear gradient from 0 to 10% B; 15.0 to 15.1 min linear gradient from 10 to 100% B; 15.1 to 25.0 min isocratic 100% B; 25.0 to 25.1 min linear gradient from 100 to 0% B; and column re-equilibration from 25.1 to 35.0 min at 0% B.

In the selected ion monitoring modes of unmodified and methylated peptides of mS100A9, pentavalent ions ([M + 5H]^5+^) were predominantly observed in both of them, with *m/z* 329.0 (unmodified) and 332.1 (*N*π-methylated), and the MRM-based transitions of unmodified and *N*π-methylated peptide were *m/z* 329.0 > 110.15 and 332.1 > 124.15, respectively. The MS operating conditions, data acquisition, and processing were the same as the aforementioned description. Based on the data obtained from the standards, calibration curves were constructed for mS100A9 C-terminal peptide analogs (unmodified and *N*π-methylated) in the range of 5.0 to 50 pmol. The observed concentrations and percentages of the analytes in each experiment were calculated from the calibration curves.

### *In vitro* MT assay

*In vitro* methylation reactions were performed with 3 μg GST-METTL9 WT or 2GA together with 3 to 5 μg substrate proteins in the presence of 1 μCi [^3^H]-SAM (PerkinElmer) in PBS buffer. After incubation at 30 °C for 2 h, reactions were resolved by SDS-PAGE, and gels were stained with Coomassie blue, followed by soaking in Amplify fluorographic reagent (GE Healthcare) for 20 min. The gels were dried under vacuum at 80 °C for 1 h and then exposed to Amersham Hyperfilm ECL (GE Healthcare) at −80 °C for 3 days.

### Fluorescent microscopy

HEK293T and HeLa cells were seeded overnight onto 35-mm glass bottom dishes (Matsunami) and then transfected with pcDNA3/mMETTL9-HA constructs using GeneJuice transfection regent (Novagen) for 24 h. Cells were fixed with 3.7% formaldehyde for 15 min at room temperature. After washing with PBS for three times, cells were permeabilized with 0.1% Triton X-100 in PBS for 10 min at room temperature, followed by blocking with immunofluorescence blocking buffer (1× PBS with 3% bovine serum albumin and 0.1% Tween-20) for 1 h. Each sample was incubated with anti-HA (12CA5) antibody (1:200) in antibody dilution buffer (1× PBS with 1% bovine serum albumin and 0.1% Tween-20) for 2 h at 4 °C. Cells were washed thoroughly with 1× PBS with 0.1% Tween-20 and then incubated for 1 h at room temperature with Alexa488 antimouse antibody (1:1000) in antibody dilution buffer. After washing with 1× PBS with 0.1% Tween-20, cells were stained with Alexa594-conjugated concanavalin A (Thermo Fisher Scientific; 50 mg/ml) as an ER marker and Hoechst 33258 (Dojindo; 1 mg/ml) as a nuclear marker for 15 min at room temperature. The fluorescent signals were acquired with an Olympus FV10i confocal microscope and processed with FLUOVIEW software (Olympus).

### Coimmunoprecipitation assay

HEK293T cells transiently expressing Flag-S100A9 together with METTL9 (WT)-HA or METTL9 (DSP)-HA were lysed in cell-lysis buffer (20 mM Hepes–KOH, pH 7.9, 150 mM NaCl, 0.1% Triton X-100, and 0.1 mM EDTA) supplemented with 1× Protease Inhibitor Cocktail (Nacalai Tesque, Inc), and Flag-tagged proteins were then immunoprecipitated with ANTI-FLAG M2 Affinity Gel. Protein G Sepharose 4B (GE Healthcare) mixed with normal mouse IgG (Sigma–Aldrich) was used as negative control. After washing thoroughly with cell-lysis buffer, the immunoprecipitates were separated by SDS-PAGE, followed by Western blot analysis using anti-Flag and anti-HA antibodies.

### GST pull-down assay

For GST-tagged proteins, GST alone and GST-S100A8 were expressed in the BL-21 *E. coli* strain and purified using Glutathione Sepharose 4B. For high-methylated and low-methylated S100A9 proteins, HEK293T cells were transfected with 5 nM of nontargeting control or *METTL9* siRNAs (Dharmacon) and incubated for 24 h. The cells were further transfected with pcDNA3/Flag-mS100A9 and then lysed in the cell-lysis buffer supplemented with 1× Protease Inhibitor Cocktail, followed by mixing with the GST or GST-S100A8 proteins immobilized on glutathione beads for 3 h at 4 °C. After washing thoroughly with the same buffer, proteins were separated by SDS-PAGE, transferred to a polyvinylidene fluoride membrane, stained with Ponceau S (Sigma–Aldrich), and probed with the anti-Flag antibody.

### RT-quantitative PCR

To prepare cDNA templates for real-time RT-PCR, total RNA was extracted from HEK293T cells using ISOGEN II (Nippon Gene). After DNase treatment, the total RNA was converted to cDNA using ReverTra Ace quantitative PCR RT Master Mix (TOYOBO) and subjected to real-time quantitative PCR analysis using TB Green Premix EX TaqII (TaKaRa Bio, Inc) on a Thermal Cycler Dice (TaKaRa Bio, Inc). The relative mRNA expression was calculated based on the delta-delta-Ct algorithm. The primer set used for quantitative PCR analysis was as follows:

Human *mettl9*_F: 5′-TTGGAGCCAACTAGAGGCAG-3′
Human *mettl9*_R: 5′-CACTTGCCACCTACGTTTTCC-3′
Human β-*actin*_F: 5′-CAAGAGATGGCCACGGCTGC-3′
Human β-*actin*_R: 5′-CTAGAAGCATTTGCGGTGGACG-3′

### *In vitro* zinc-binding assay

Zinc-chelating chromatography was performed as previously described (34). Briefly, the iminodiacetic acid-resin-based iminodiacetic acid-Sepharose column (Hi-Trap chelating; Cytiva) equilibrated with zinc sulfate (ZnSO_4_) at a final concentration of 100 mM. After several washes, the S100A9 peptides (amino acids 98–113) either nonmethylhistidine or *Nπ*-methylhistidine at His-107 were loaded on the columns at 0.45 nmol concentration, and binding was allowed to proceed for 1 min at room temperature, followed by washing out the unbound peptides. After extensive washes, the bound peptide was eluted with 50 mM EDTA. The peptide concentration of both eluted fractions was measured by LC–MS/MS mentioned previously. The same experiments were repeated three times independently.

## Data availability

All data are contained within this article. Reagents and plasmids described in this article are available upon request.

## Supporting information

This article contains supporting information.

## Conflict of interest

The authors declare that they have no conflicts of interest with the contents of this article.
