# Supplementary material for: siRNA screening identifies METTL9 as a histidine Nπ-methyltransferase that targets the proinflammatory protein S100A9
Source: J Biol Chem. 2021 Sep 23;297(5):101230. doi: 10.1016/j.jbc.2021.101230 (PMC8571522; doi:10.1016/j.jbc.2021.101230)
Supplement: Figures S1–S6 and Table S1 [file mmc1.pdf]

siRNA screening identifies METTL9 as a histidine N^{π}-methyltransferase that targets the pro-inflammatory protein S100A9

Hiroaki Daitoku*, Momoka Someya, Koichiro Kako, Takahiro Hayashi, Tatsuya Tajima, Hikari Haruki, Naoki Sekiguchi, Toru Uetake, Yuto Akimoto, Akiyoshi Fukamizu*

*Hiroaki Daitoku and Akiyoshi Fukamizu
Email: hiroakid@tara.tsukuba.ac.jp and akif@tara.tsukuba.ac.jp

This PDF file includes:

Figures S1 to S6
Table S1

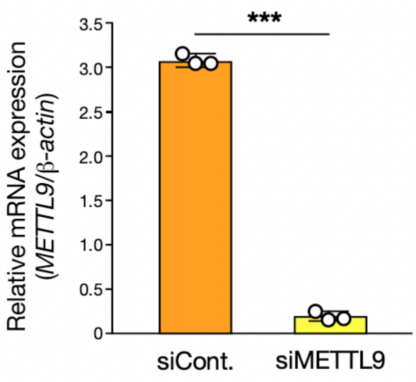

Figure S1. siRNA knockdown of *METTL9* markedly reduces its mRNA. HEK293T cells were transfected with either control or *Mettl9*-specific siRNAs. The mRNA levels of *Mettl9* were measured by qPCR analysis and normalized against the mRNA levels of *β-actin*. Mean ± s.d. (*n*=3 independent experiments). ***P<0.001; two-tailed Student's *t* test.

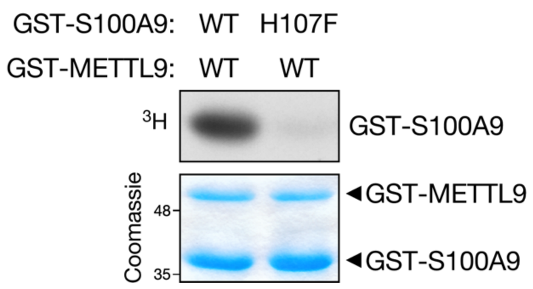

Figure S2. METTL9 specifically methylates S100A9 at His-107 *in vitro.* GST-S100A9 wild-type or H107F mutant was incubated with GST-METTL9 in the presence of ^{3}H-SAM. The incorporation of [^{3}H]-methyl into proteins was visualized by autoradiography (*Top*). Coomassie blue staining of proteins used in the reaction is shown as a loading control (*Bottom*).

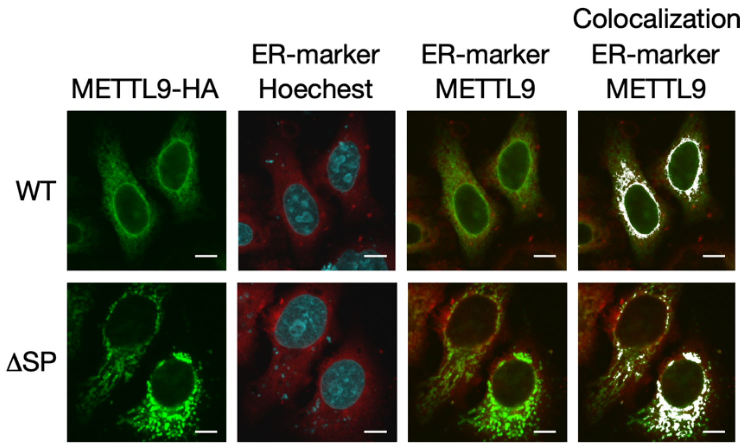

Figure S3. METTL9 localizes to the ER in HeLa cells. Representative immunofluorescence images of METTL9 wild-type (*Top*) or ΔSP mutant (*Bottom*) localization in HeLa cells. Green, METTL9-HA; red, ER-marker (Alexa594-conjugated Concanavalin A); blue, nucleus (Hoechst33258); scale bar, 10 μm. Images of ER-marker were merged with Hoechst and METTL9-HA staining. Colocalization indicates overlay of the ER-marker and METTL9-HA, shown as white pixels.

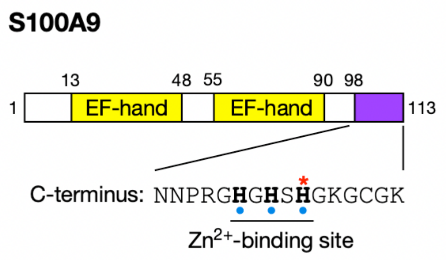

Figure 4. A schematic model of mouse S100A9.

Top, domain structure of S100A9 containing the two EF-hand (yellow) and a C-terminal Zn^{2+}-binding site (purple). *Bottom*, the C-terminal sequence of mouse S100A9. Histidine residues involved in zinc coordination are marked with blue spheres, and methylated histidine at 107 is indicated with a red asterisk.

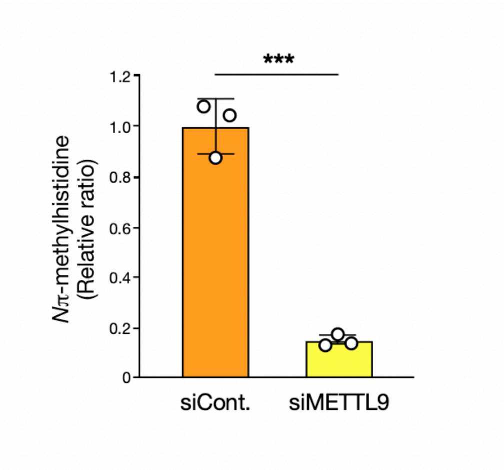

Figure S5. siRNA knockdown of *METTL9* reduces *N*π-methylhistidine levels of Flag-S100A9. HEK293T cells transfected with control or METTL9 siRNAs were further transfected with Flag-S100A9 expression plasmid, followed by immunopurification and acid hydrolysis. Their methylhistidine contents were determined by LC-MS/MS and shown as a relative ratio compared to control siRNA knockdown. Mean ± s.d. (n=3 independent experiments). ****P*<0.001; two-tailed Student's *t* test.

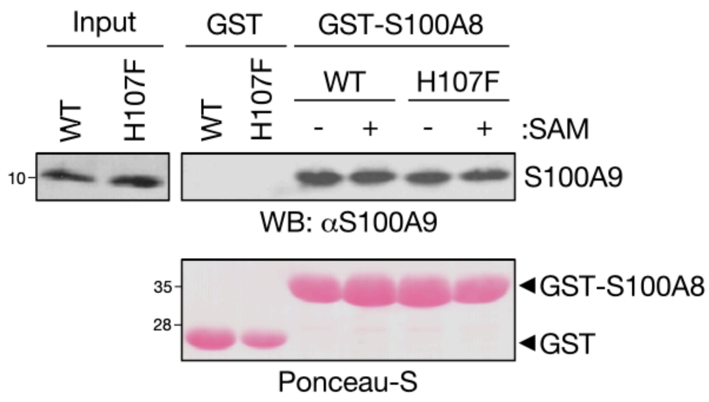

Figure S6. METTL9-mediated methylation of S100A9 has no effect on its heterodimer formation with S100A8.

Recombinant S100A9 wild-type and H107F proteins were methylated by GST-METTL9 in the absence or presence of SAM and then subjected to GST pull-down assay with GST alone or GST-fused S100A8, followed by Western blotting using an anti-S100A9 antibody (*Top*). The input indicates 5% of whole-cell lysates used for the GST pull-down assay. Ponceau-S staining of GST proteins used in the reaction is shown as a loading control (*Bottom*).

Supplementary Table S1: The sequences of siRNA pools used in the screening.

Gene Symbol	Gene Accession	Sequence
Non-targeting Control		UGGUUUACAUGUCGACUAA
		UGGUUUACAUGUUGUGUGA
		UGGUUUACAUGUUUUCUGA
		UGGUUUACAUGUUUUCCUA
METTL18	NM_033418	GAUUAUAACAGUAUGGUGA
		GCAUGUUAAGUUAUCAGUA
		GAUCACUUGUGGGAACAUA
		GUUUAUGAGGGAGGCUUAA
METTL20	NM_001135864	CGCUGAAGGAAUAUGAUUA
		CUAUAGAACUCGAGUACUG
		GAGGACAACUGUGAUGUAA
		CCUUGUUGUUCUUGGCGAU
METTL21A	NM_145280	AGUGUGAGAUUUCGAUUUA
		UGACUAUCACGGAUCGAAA
		AUGAGGAGACCACGGAAUU
		CCUGAAAUCUGGCCGGAUG
METTL21B	NM_015433	GUGCAAUUAUUUCGAGAGU
		GAGCCUUGAUCACGGAACA
		GGCAAGAAGGUGAUCGAAC
		CUUCAGUCCAUUCCGCAAA
METTL21C	NM_001010977	GGGCAAACAAAUUCAGGUU
		GCUAUGGCUUUGUGUCAAU
		ACUUGAAAUUGGUGCCGGA
		GGAUUGAGAAGGUAGUGCA
METTL21D	NM_001040662	GCUCUAACAACCUGGGUAA
		UGGUACAGUGCUACGACUA
		CUAAAAGAUAUCAGCGGAU
		CAGCUUAGGUUACUUAGUU
METTL22	NM_024109	CAUGUGAAGCCUACGAUCA
		CAAGGCAGCCCGCACGAUA
		UAGCACAGGAAGAAGACGA
		CCGAAGUGUUUUACGACGA
METTL23	NM_001080510	AAGGACAGUCUCUGAAUUA
		GGACUAACAUGGGGUCAUA
		UUGAGAAUGCAGUGGGUCU
		UCACCUAGACAACACUUAA
METTL25	NM_032230	GGGAGGAGCUGGUCGACUU
		UGACUUGGGUUCCGGUAAA
		AUAAUGAACUACUACGAGA
		GUUGUUUGAUCCCGUGAAA
CAMKMT	NM_024766	GCAUAAUAGUGGAUCCUUG
		CCGGACAUAUAUGAAGAAA
		CGCCGAGGGAAUACUUUAA
		GGUGCAUGGGUCCAAUAUA
EEF2KMT	NM_201598	GAAAUCAAGGCCAACCAAA
		GCCUCUACGUGCUGCCCAU
		GCACGGCCAUCAUCUCCUA
		GUGCUGCUCGAAAGGGUUU
FAM86B1	NM_001083537	AAUCAAAACUGCCCUCUAA
		GAGCAGAGCUUAACUCAUU
		UGGAAUGUUUGGAGAUUAU
		CAUGAAGAACAGAAGACAG
FAM86B2	XM_928336	GGGCAUACAUCUUCAGCGA
		CAUUAGAGGCAGACAUCAC
		CCGGCAGCCUUCAUUAACA
		GCCAUCAUCUCCCACGGUA
FAM86C1	NM_152563	GAGACCAAAUGCAGGGUAA
		CUAAUAAGACAUCGGCUAU
		GAGCAGAGCUUACUUCAUU
		ACUGGAAUGUUUGGAGAAU
CARNMT1	NM_152420	UCUUGUUUGUGGUCCGUAA
		CUAAGAAAACUAUGCGUAA
		AGGAGAGGCUUGAGCGUGA
		UAGCAUCACAGGCCGGUAA
HNMT	NM_001024074	CGGGAAAUAUGUUGAAUCU
		CCACGGAACACCAGUGCAU
		CAUCUUCCAUGAGGAGCUU
		AGGAAUUCAUGGACAAGAA
METTL6	NM_152396	UAGAAGAAGAUCCGAAUAU
		AGAAGUGGUAAACGAGUAU
		UCCAAGAGCCAUUGAAUAU
		GGAUGUUGUUAUGUUGAUA
METTL9	NM_016025	AUAAACACGUGGAGGUCGA
		UGGGUAUAAAGGCGAAUUA
		GUGAAGGCGACAUGUAUAA
		AGAGUUGGGUUAUGUGAUU
SETD3	NM_199123	GGUAAGAAGAGUCGAGUAA
		GCUAAUGACUGUUGAAUCU
		GCACUGGCCUUUCAUUUGC
		ACACUCCUCUCUACUUUGA
SETD4	NM_001007259	GUUACGGCCCUCACGAUAA
		GAAACUCAUUCUUACGAAA
		CCAGGUAAAAGCAGCGUUU
		GAGCCCACGUGCAGGAGUU
SMYD1	NM_198274	GUGAUUAACUGCAACGGUU
		AAGAAUGAAUGUUCGGCCA
		GUAUCAUGAGGUUGUGAAA
		GCCCAACUGUACUGUCAUA
